# Supplementary material for: Causal Relationship between Aging and Anorexia Nervosa: A White-Matter-Microstructure-Mediated Mendelian Randomization Analysis
Source: Biomedicines. 2024 Aug 16;12(8):1874. doi: 10.3390/biomedicines12081874 (PMC11351342; doi:10.3390/biomedicines12081874)
Supplement: Supplementary file 1 [file biomedicines-12-01874-s001.zip › Supplementary Figure/Figure S9/Leaveoneout plot of MR analysis for ubm-b-1929 to AN.pdf]

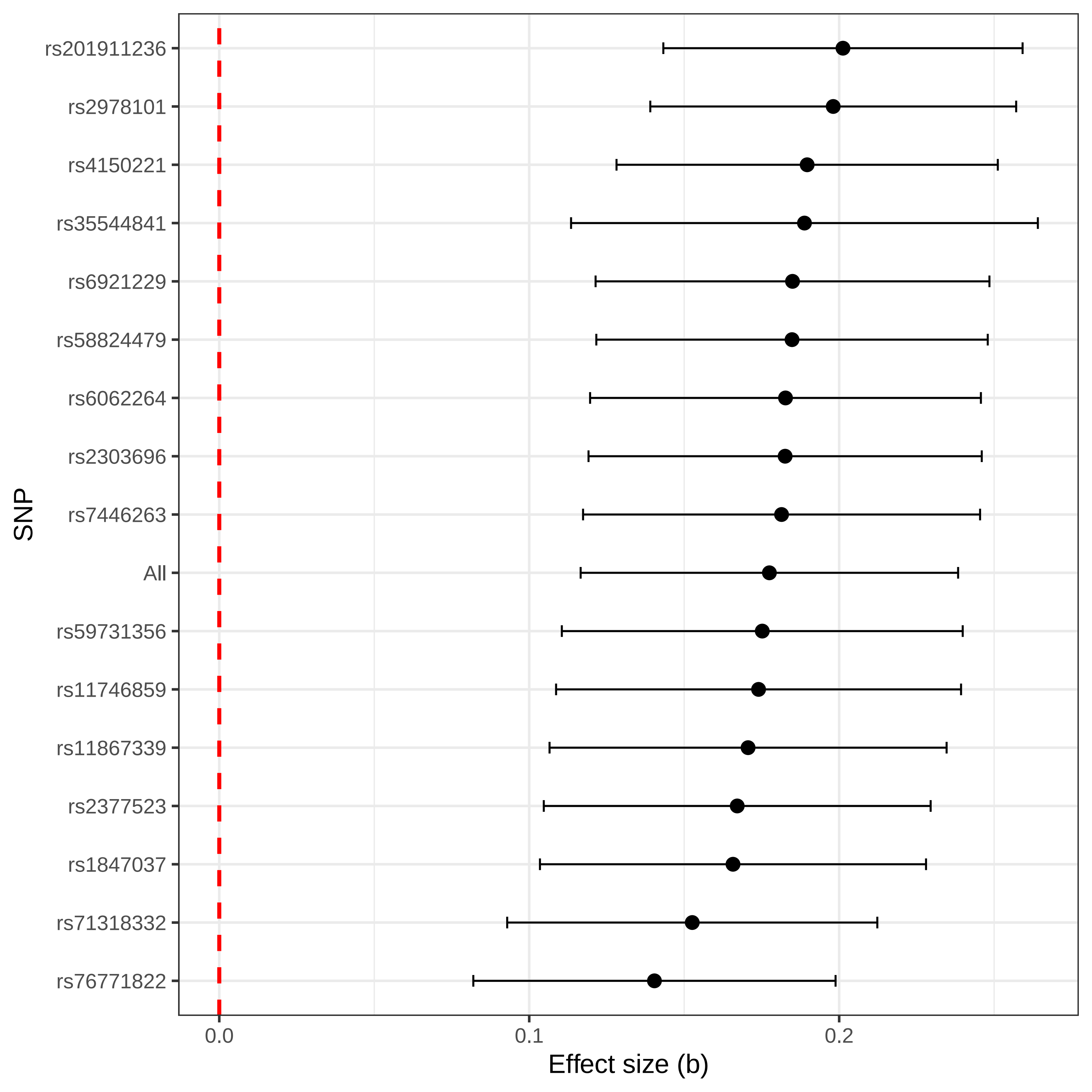
rs201911236
rs2978101
rs4150221
rs35544841
rs6921229
rs58824479
rs6062264
rs2303696
rs7446263
All
rs59731356
rs11746859
rs11867339
rs2377523
rs1847037
rs71318332
rs76771822
SNP
0.0
0.1
0.2
Effect size (b)
